# Temporal patterns of alcohol consumption and attempts to reduce alcohol intake in England

**DOI:** 10.1186/s12889-016-3542-7

**Published:** 2016-09-01

**Authors:** Frank de Vocht, Jamie Brown, Emma Beard, Colin Angus, Alan Brennan, Susan Michie, Rona Campbell, Matthew Hickman

**Affiliations:** 1 NIHR School for Public Health Research, Bristol, United Kingdom; 2 School of Social and Community Medicine, University of Bristol, Canynge Hall, 39 Whatley Road, Bristol, BS8 2PS UK; 3 Department of Clinical, Educational and Health Psychology, University College London, London, UK; 4 ScHARR, School of Health and Related Research, University of Sheffield, Sheffield, UK

## Abstract

**Background:**

The Alcohol Toolkit Study (ATS) is a monthly survey of approximately 1700 adults per month aged 16 years of age or more in England. We aimed to explore patterns of alcohol consumption and motivation to reduce alcohol use in England throughout the year.

**Methods:**

Data from 38,372 participants who answered questions about alcohol consumption (March 2014 to January 2016) were analysed using weighted regression using the R *survey* package. Questions assessed alcohol consumption (AUDIT-C) and attempts to reduce consumption.

**Results:**

Sixty-seven percent of participants reported using alcohol, with a small negative trend of about 2 % reduction over 12 months in the studied period (*P* < 0.01). These include ~25 % higher risk drinkers and ~10 % regular binge drinkers. About 20 % of higher risk drinkers indicated they were attempting to reduce their alcohol consumption. Attempts were lowest in December (−20 %; 95 % CI 0–35 %), but increases significantly in January (+41 %; 95 % CI 16–73 %) compared with other months (*P* < 0.001), indicating a small net gain; at least in attempts to reduce. However, there was no evidence that the increased motivation in January was accompanied by a reported decrease in consumption or binge drinking events. This could be an artefact of the use of AUDIT questions, but could also reflect a disconnect between attempting to reduce alcohol consumption and subsequent change; maybe as a result of lack of continuing support.

**Conclusions:**

January is associated with moderate increased attempts to reduce alcohol consumption. However, we find little evidence of a change in alcohol consumption. In part, this may be due to temporal insensitivity of the AUDIT questions.

**Electronic supplementary material:**

The online version of this article (doi:10.1186/s12889-016-3542-7) contains supplementary material, which is available to authorized users.

## Background

Hazardous alcohol consumption results in a considerable burden for society [1], and is recognized as a major public health problem in the United Kingdom (as well as in other countries) [2].

A seasonal rise in alcohol consumption over the December festive period have been shown for decades [3–7], but it is less clear if there are consistent patterns across the rest of the year [8]. It is important to capture temporal patterns in population alcohol consumption to get an overview of when and how drinking occurs at the population level and decide if, and what kind of interventions may be required. At the same time, for methodological reasons, it is important to be aware of seasonality in consumption because estimates of annual alcohol consumption based on data from a restricted time period are likely to be biased in the presence of short-term temporal variability. However, measuring consumption is not straightforward; studies asking about consumption over longer time periods, typically over a period from several weeks up to a year, may not reflect sporadic heavy drinking occasions, while studies reflecting shorter time periods, although closer to actual consumption, may not represent “typical” consumption patterns at the individual level [9, 10].

To mitigate the burden on societies as a result of alcohol consumption [1], reduction in population consumption, especially from hazardous levels, is important. At an individual level, however, prior to the actual reduction in consumption, a willingness to change resulting in an actual attempt to reduction has to be apparent and this has to remain long enough to facilitate behavioural change. It has been shown in primary care patients with unhealthy alcohol use that motivation to change can lead to reduced consumption [11], but also that this motivation is not necessarily accompanied by actual change in consumption [12]. In the general population, however, there is a paucity of information about motivation and attempts to reduce alcohol consumption, and no data are available about temporal patterns in these. These data would be useful to maximize the effect of interventions, such as “Dryathlon” and “Dry January” in the UK, in which participants are challenged to give up alcohol for (at least) the 31 days of January [13] by exploiting knowledge on existing motivational patterns to maximize participation. For example, “Stoptober” was a mass media campaign developed to increase motivation to quit smoking and provide active wide ranging support during a 28 day period in October on the basis that this had previously been a comparatively fallow period for quitting attempts in England. By focussing on motivation and ongoing support the campaign led to a 50 % increase in quit attempts in 2012 compared to previous years [14]. Additionally, data on the association between attempts to change and actual measureable changes in alcohol consumption are important in order to evaluate the effect of interventions aimed at making people reduce their consumption and further indicate the need to improve the theory behind population-level intervention programmes.

Many studies assessing seasonality have been conducted outside of the UK, but because seasonality may differ between countries, for example because of outdoor temperatures and differences in culture, there is a need for country-specific research. Moreover, this may also change over time, and therefore we analysed data on self-reported alcohol consumption within the ‘The Alcohol Toolkit Study’ (ATS) to evaluate contemporary seasonal patterns of alcohol consumption in England. Additionally, the ATS data were used to assess monthly patterns in attempts to reduce alcohol consumption, providing the first data on this for the general population.

## Method

### Data source and study population

The analyses presented here are based on data from a monthly cross-sectional population survey: ‘The Alcohol Toolkit Study’ (ATS), a sister survey to the Smoking Toolkit Study (STS) which has been collecting data since 2006 [15]. The protocol of the ATS is described in detail by Beard *et al*. [16]. In short, the ATS is a cross-sectional household survey conducted monthly by a UK market research organisation which aims to collect computer-assisted household interviews of approximately 1700 adults per wave aged 16 years of age or older in England. The first wave was conducted in March 2014 and at the time of writing 23 waves have been completed, resulting in data from 38,624 participants. Data are collected using a hybrid between random probability and simple quota sampling (random location sampling), in which England is split into 171,356 ‘Output Areas’ of about 300 households each and which are subsequently stratified based on socio-economic profile (using ACORN methodology [17]) and geographic region, and further divided into 17 groups and 56 types based on census and lifestyle survey data. Areas are randomly allocated to interviewers who then visit households within the locality starting at a random point in the area and conduct electronic interviews with one member of a household until interviewers achieve quotas specified on the likelihood of possible respondents being at home are fulfilled. Although a response rate for this methodology cannot be calculated because interviewers choose which property(/ties) to approach in each small output area to reach their quota (in contrast to random probability sampling in which the response can be registered at each allocated address), this form of location sampling is generally considered superior to conventional quota sampling because the impact of selection of properties is significantly reduced by the random allocation of small output areas to interviewers [18].

The ATS computer-assisted interviews address prevalence and frequency of alcohol consumption using the ‘Alcohol Use and Disorders Identification Test (AUDIT)’ questionnaire [19–21], augmented with a range of questions relating to alcohol consumption and attempts to cut down, as well as data on important personal and demographic factors (described in detail in [16]). An AUDIT score of 8 or AUDIT-C score of 5 was used as a cut-off to indicate higher risk alcohol consumption, and these participants were further questioned about their willingness to reduce consumption.

Reporting of typical alcohol consumption is directly influenced by recent drinking behaviour [8, 22] and we consider AUDIT questions 1 to 3 to be indicative of respondents’ current consumption patterns, and use these to calculate four measures of exposure:

1. AUDIT question 1 (How often do you have a drink containing alcohol?) was used as an indication of current frequency of alcohol consumption events. This was further dichotomized to indicate abstainers (*e.g.* at least monthly or less) and those that drink alcohol.
2. AUDIT question 2 (How many alcohol units do you have on a typical day when you are drinking?) was used as an indication of average quantity of alcohol consumption. This was registered from 1 (non-drinker) to 7 (16 or more drinks).
3. AUDIT question 3 (How often did you have six or more standard drinks on one occasion?) was used to infer episodic or regular binge drinking (at least weekly).
4. AUDIT questions 1 and 2 were also multiplied to derive a semi-quantitative measure of cumulative consumption.

### Statistical analysis

Prior to statistical analyses, the data were weighted using a rim (marginal) weighting adjustment involving an iterative sequence of weighing adjustments based on nationally representative target profiles for gender, working status, number of children, age, social-grade and geographical region.

Data were analysed using the *survey* package in *R* (version 3.2) with weighted (quasibinomial) regression models specified using the *svyglm* command. Non-binary and Gaussian outcomes were analysed using weighted ordinal regression from the *svyolr* command and generalised linear models from the *svyglm* command, respectively. The month March was used as the (arbitrary) reference month because March 2014 data were the first data available. The distribution of cumulative consumption was right-skewed and was therefore log(e)-transformed prior to analysis to resemble Gaussian distributions.

Temporal autocorrelation was evaluated using the Breusch-Godfrey test and graphically based on (partial) autocorrelation function plots (ACF/PACF), which indicated no significant serial autocorrelation (BG test up to order 3; *P* = 0.85). ACF and PACF plots are provided in Additional file 1.

Sensitivity analyses were conducted using ATS respondents’ self-reported ‘alcohol expenditure’ and UK alcohol sales data from Her Majesty’s Revenue and Customs (HMRC) [23].

## Results

Demographics of the weighted and unweighted samples are shown in Table 1. A total of 38,624 participants were interviewed in 23 months that the ATS has been running (*e.g.* an average of 1679 per month) of which 38,372 provided data on alcohol consumption (99 %). The impact of weighing is relatively minor across most variables, indicating that distributions of age of participants, life stages and geographical regions were representative of the English population, but weighting adjusted for some oversampling of unemployed and working class participants compared to those from the middle class. The weighted sample produced comparable proportions of male (49 %) and female (51 %) participants, with the majority being Caucasian (86.5 %; (95 % confidence interval (CI) 86.2–86.9 %), and with all regions in England represented. A representative distribution across age categories was also included, ranging from 14.0 % (95 % CI 13.7–14.4) of 55–64 year olds to 20.8 (95 % CI 20.4–21.3) of those aged 65 and over. Most participants were post-family (47.9 % (95 % CI 47.9–49.0) with only 6.3 % (95 % CI 6.0–6.7) pre-family. Distribution across social classes declined from 27 % (95 % CI 26.9–27.9) in the middle class to 9.5 % (95 % CI 8.2–8.7) unemployed.

**Table 1:** Raw and weighted demographics

	Raw data				Weighted sample					
	All participants		Higher Risk Drinkers		All participants			Higher Risk Drinkers		
	N	%	N	%	N	%	95 % CI^f^	N	%	95 % CI^f^
Complete Sample					38,624	100 %				
Missing data alcohol consumption					252	0.7 %				
Sample	38,624	100 %			38,372	99.3 %		10,334	26.9 % of total	
Higher risk drinkers^a^	9717	25.2 %	9717	100 %	10,334	26.9 %	26.4–27.4	10,334	100 %	
Sex (female)	18,777	48.6 %	3380	34.8 %	19,588	51.0 %	50.5–51.6	3705	35.8 %	34.8–36.9
Ethnicity (Caucasian)^b^	31,557	81.7 %	9252	95.2 %	33,080	86.5 %	86.2–86.9	9940	96.6 %	96.2–96.9
Age (16–24 years)	6136	15.9 %	2072	21.3 %	5490	14.3 %	13.9–14.7	1928	18.7 %	17.9–19.4
25–34	5889	15.2 %	1414	14.6 %	6422	16.7 %	16.3–17.1	1732	16.8 %	15.9–17.6
35–44	5654	14.6 %	1457	15.0 %	6417	16.7 %	16.3–17.1	1816	17.6 %	16.7–18.4
45–54	5720	14.8 %	1721	17.7 %	6674	17.4 %	16.9–17.8	2107	20.3 %	19.5–21.3
55–64	5774	14.9 %	1618	16.7 %	5394	14.0 %	13.7–14.4	1565	15.1 %	14.4–15.9
65+	9199	23.8 %	1435	14.8 %	8005	20.8 %	20.4–21.3	1187	11.4 %	10.9–12.1
Life stage (single)^c^	5665	14.7 %	2120	21.8 %	5443	14.2 %	13.8–14.6	2133	20.6 %	19.8–21.5
Pre-family	2069	5.4 %	680	7.0 %	2408	6.3 %	6.0–6.7	837	8.1 %	7.5–8.7
Family	11,330	29.3 %	2441	25.1 %	11,925	30.6 %	30.6–31.6	2856	27.6 %	26.7–28.6
Post family	19,259	49.9 %	4466	46.0 %	18,575	47.9 %	47.9–49.0	4502	43.6 %	42.5–44.7
NRS Social Grade^d^										
AB	8131	21.1 %	2237	32.0 %	10.403	27.1 %	26.6–27.6	3304	32.0 %	30.9–33.0
C1	11,854	30.7 %	2034	29.1 %	10,525	27.4 %	26.9–27.9	3025	29.3 %	28.3–30.2
C2	7962	20.6 %	1594	22.8 %	8438	22.0 %	21.5–22.4	2235	22.5 %	21.6–23.4
D	6123	15.9 %	723	10.3 %	5782	15.1 %	14.7–15.4	1075	10.4 %	9.8–11.0
E	4302	11.1 %	406	5.8 %	3255	9.5 %	8.2–8.7	606	5.9 %	5.4–6.3
Government Region^e^										
East Midlands	3078	8.0 %	626	6.4 %	3334	8.7 %	8.4–9.0	723	7.0 %	6.4–7.6
Eastern	3468	9.0 %	740	7.6 %	4319	11.2 %	10.9–11.6	992	9.6 %	8.9–10.3
London	7176	18.6 %	1018	10.5 %	5648	14.7 %	14.4–15.0	885	8.6 %	8.0–9.1
North East	1986	5.1 %	837	8.6 %	1945	5.1 %	4.8–5.3	851	8.2 %	7.7–8.8
North West	5655	14.6 %	1883	19.4 %	5081	13.2 %	12.9–13.6	1748	16.9 %	16.2–17.7
South East	4307	11.2 %	1302	13.4 %	6248	16.3 %	15.8–16.7	1937	18.7 %	17.8–19.7
South West	3431	9.9 %	803	8.3 %	3950	10.3 %	9.9–10.6	1004	9.7 %	9.0–10.4
Welsh border	74	0.2 %	12	0.1 %	66	0.2 %	0.1–0.2	12	0.1 %	0.0–0.2
West Midlands	4429	11.5 %	804	8.3 %	3898	10.2 %	9.8–10.5	754	7.3 %	6.8–7.8
Yorkshire and the Humber	4767	12.3 %	1692	17.4 %	3907	10.2 %	9.9–10.5	1428	13.8 %	13.2–14.5

^a^AUDIT score ≥ 8 or AUDIT-C score ≥ 5

^b^missing *n* = 178

^c^Single is up to age 39, not married, and no children in household; Pre-family is aged up to 39, married or living with partner, no children in household; Family means children in household; Post-family is aged 40 and above and no children in household (missing *n* = 49)

^d^NRS social grades: AB(upper middle and middle class), C1 (lower middle class), C2 (skilled working class), D (working class), E (non working)

^e^missing: *n* = 1

^f^95 % confidence limits

**Fig. 1:**
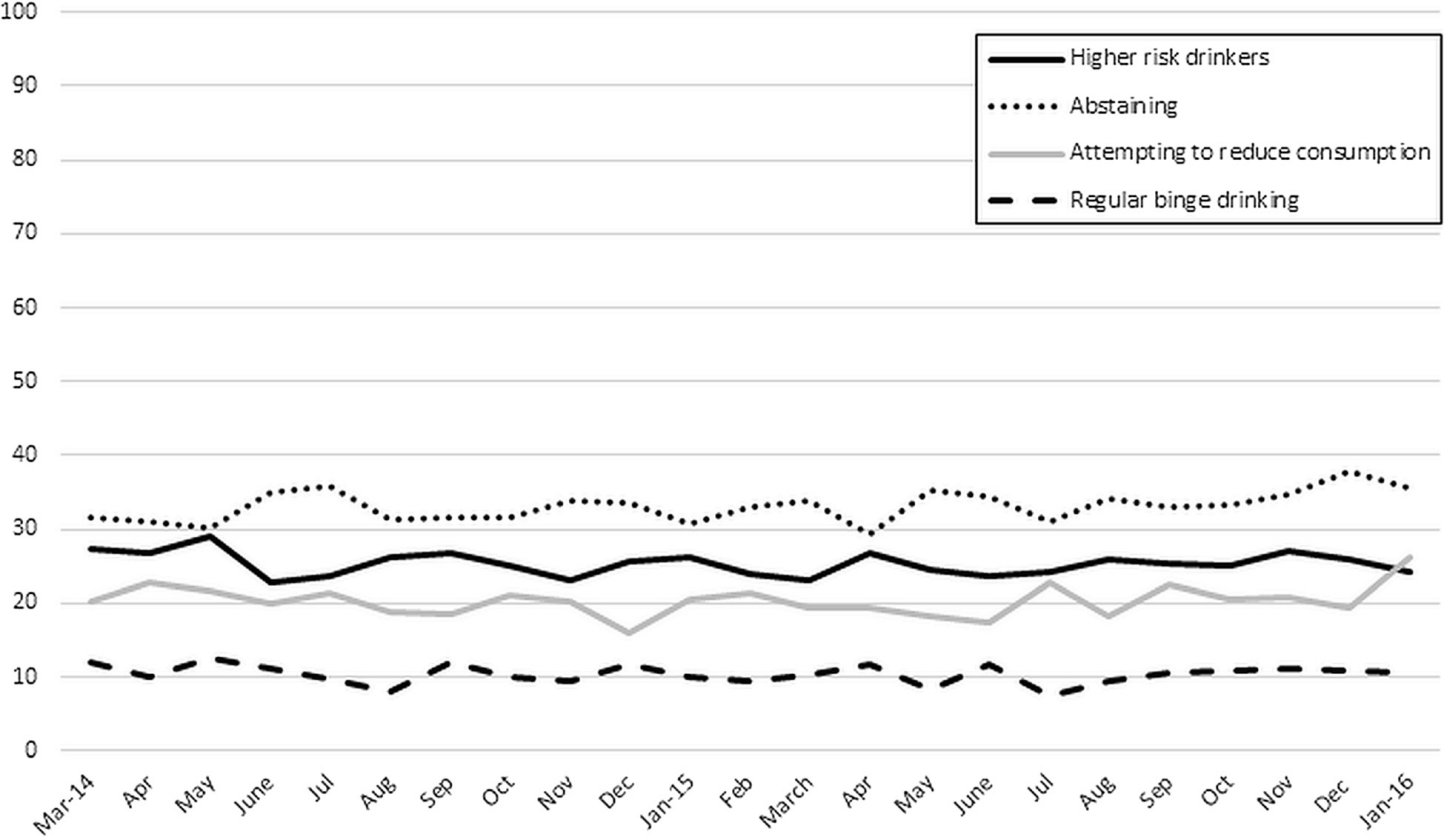
Unweighted population prevalence of respondents abstaining from alcohol consumption, higher risk drinkers, regular episodic or binge drinkers and attempts to cut down alcohol consumption (amongst higher risk drinkers) in sample. Abstaining and higher risk drinking answered by ~1700 respondents per wave; regular binge drinking answer by ~66 % of respondents; attempting to reduce alcohol consumption answered by ~26 % of respondents

Trends in alcohol consumption pattern and attempts to cut down alcohol consumption over the measured time period are shown for the unweighted data in Fig. 1. The average population prevalence of abstainers is 33.1 % (95 % CI 32.6–33.6), 25.3 % (95 % CI 24.9–25.8) are higher risk drinkers, 10.4 % (95 % CI 10.0–10.8) of respondents regularly binge drink, and 20.4 % (95 % CI 19.6–21.2) of the population are attempting to reducing their alcohol consumption.

**Fig. 2:**
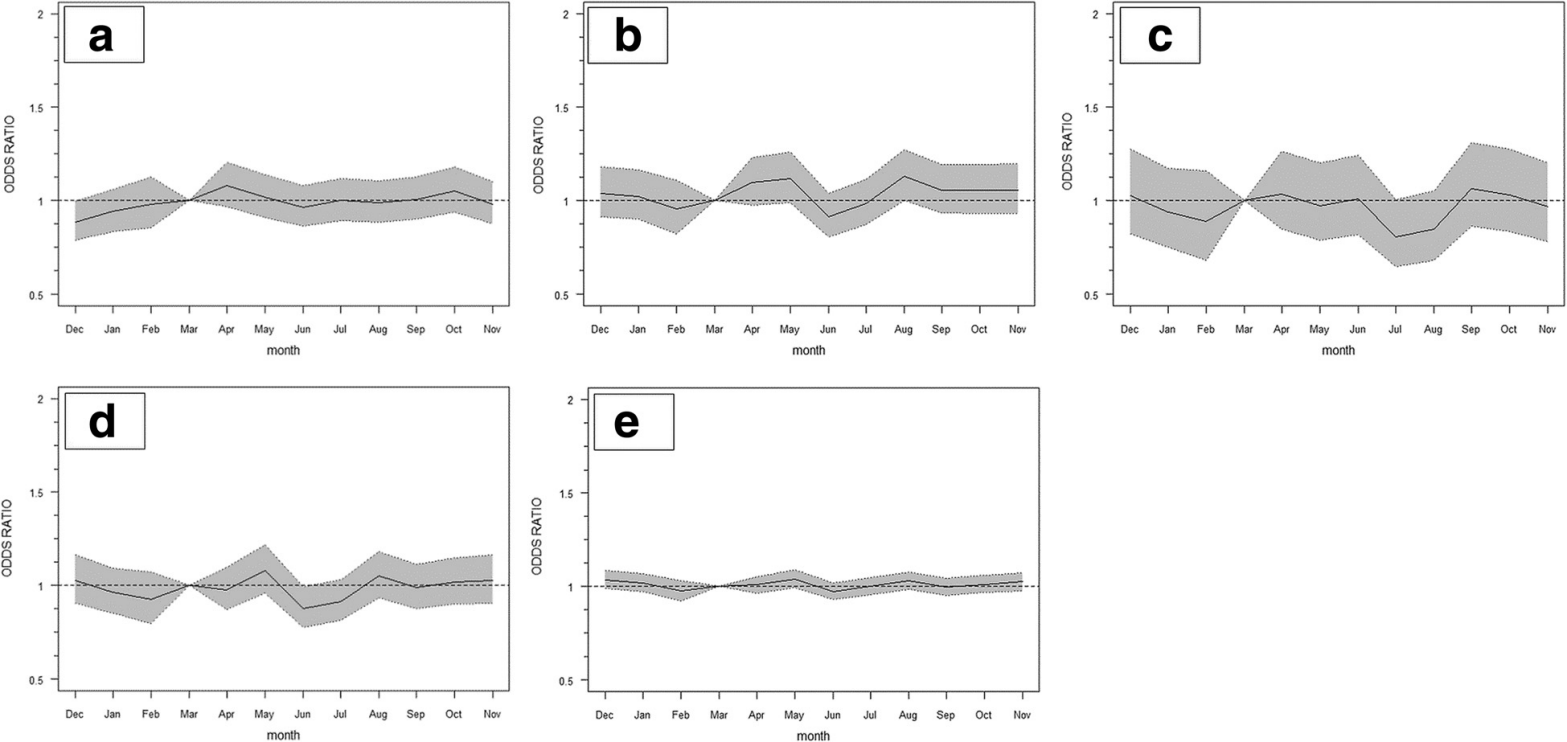
Odds ratio (relative to March, adjusted for time trend) and 95 % confidence interval for (**a**) alcohol drinkers, (**b**) higher risk drinker (AUDIT-C score of 5+) (August (*P* = 0.05)), (**c**) regular episodic or binge drinking (July (*P* = 0.05)), (**d**) increased average number of drinks on a typical drinking day (June (*P* = 0.04)), and (**e**) cumulative alcohol consumption

Differences in alcohol consumptions patterns throughout the year, as estimated from the AUDIT questions, are relatively stable (Fig. 2a-e) and although they indicate lower consumption and less binge drinking in early to mid-summer—depending on the metric—differences are non, or only borderline, significant.

25.3 % (*N* = 9717) of participants were classified as higher risk drinkers (relatively stable at *N* = 400–450 per month), and these were asked additional questions about attempts to cut down their alcohol consumption. This resulted in a post-weighting population prevalence of higher risk drinking of 26.9 %. High risk drinkers were more often male, Caucasians, post-family, middle class and from the North-West or South-East of England, and less often from the West Midlands (Table 1).

**Fig. 3:**
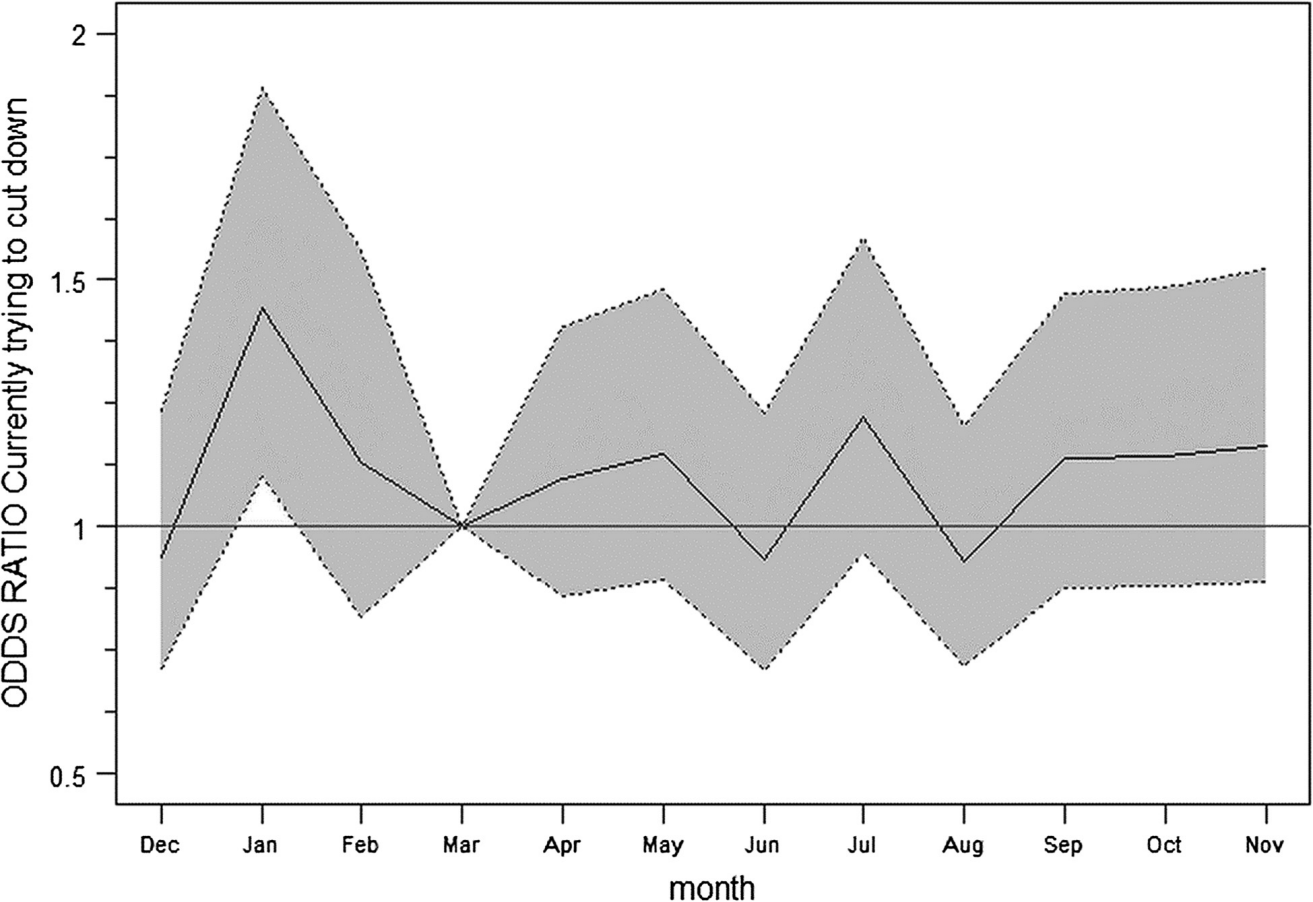
Odds ratio and 95 % confidence interval for currently trying to cut down alcohol consumption relative to reference month March. *Model adjusted for time trend. Motivation to cut down consumption significantly higher in January (P < 0.001)*

Attempts to reduce alcohol consumption are also relatively stable across the year, with the exception of January during which there is a significantly higher (P ~ 0.006) motivation to cut down alcohol consumption (Fig. 3).

In fact, the weighted data indicate a 45 % (OR 1.45; 95 % CI 1.11–1.90) higher probability of participants attempting to cut down consumption in January compared to the reference month, or, equivalent, 36 % (95 % CI 12–64 %) higher relative to any other month of the year (Table 2). Quantitatively, this corresponds to a 5 % (95 % CI 2–9 %) higher population prevalence compared to the rest of the year. An opposing effect on attempts, although about half the size and not reaching statistical significance, can be observed in December. There are no significant differences in reported motivation between different age groups, sexes, social classes, educational groups, or occupational groups, with the noticeable exception of retired people who were more likely to report to be trying to reduce consumption (Table 2). The observed effect for January is stable over time with a 41 % increase in attempts to reduce consumption in January 2015 and a 38 % increase in January 2016, respectively (Additional file 1: Table S1). Additional file 1: Table S1 further indicate reduced motivation in December and in August, indicating that motivation to reduce alcohol consumption in January (and to a lesser extent December and August, which are not statistically significant after Bonferroni correction) are unique.

**Table 2:** Changes in motivation to reduce alcohol consumption and in consumption of alcohol in January and December

	Motivation to reduce alcohol consumption					
	Model 1^a^		Model 2^a^		Model 3^b^	
	OR	95 % CI	OR	95 % CI	OR	95 % CI
Reference (other months)	1	-	1	-	1	-
January	1.36	1.12 – 1.64	1.33	1.10–1.61	1.38	1.13–1.70
December			0.85	0.69–1.05	0.85	0.68–1.05
Differential motivation sub-groups (model 3 plus interactions)						
Interaction			*P*-value^c^			
Age (6 categories)			0.76			
Sex (2 categories)			0.53			
Social class (5 categories)			0.29			
Occupation (7 categories)			0.16			
Education (9 categories)			0.85			
Alcohol consumption indicators						
	OR	95 % CI	OR	95 % CI	OR	95 % CI
Any alcohol consumption^d^						
reference (other months)	1	-	1	-	1	-
January	0.95	0.88–1.04	0.94	0.86–1.02	0.92	0.84–1.01
December			0.88	0.81–0.96	0.86	0.79–0.95
Average number of drinks per occasion^e^						
reference (other months)	1	-	1	-	1	-
January	0.97	0.88–1.06	0.97	0.89–1.07	0.94	0.85–1.04
December			1.04	0.95–1.14	1.04	0.0.94–1.15
Higher risk drinking^d^						
reference (other months)	1	-	1	-	1	-
January	0.98	0.90–1.08	0.98	0.90–1.08	0.96	0.87–1.06
December			1.00	0.91–1.10	0.99	0.90–1.06
Regular, at least weekly, binge drinking (>6 drinks per occasion)^d^						
reference (other months)	1	-	1	-	1	-
January	0.97	0.82–1.14	0.97	0.83–1.15	0.92	0.77–1.11
December			1.06	0.90–1.25	1.05	0.88–1.25
Cumulative consumption^f^						
reference (other months)	1	-	1	-	1	-
January	1.01	0.97–1.04	1.01	0.98–1.04	1.00	1.00–1.00
December			1.03	0.99–1.06	1.02	0.99–1.03

^a^also adjusted for time to account for linear in/decreases over time

^b^adjusted for time, age, sex, social class, education and occupation

^c^Statistically significant (*P* < 0.05) difference in motivation for any of the interactions was only observed for retired participants (OR = 2.78 (95 % CI 1.12–6.36))

^d^quasibinomial regression model

^e^ordinal regression model

^f^generalized linear model

The increase in attempts to cut down on alcohol consumption does not, however, seem to be accompanied by a decrease in the consumption of alcohol, nor in a reduction of the proportion of people who binge drink weekly or more (Table 2). We further observe that the proportion of people reporting that they consumed alcohol in December was significantly lower, which is not supported by other measures of consumption, but is also not indicative of the festive season. Analyses of average drinking (*e.g.* number of standard drinks on one occasion (AUDIT question 2)) indicated this was relatively stable across the year at about 2 drinks on average, with an ordinal logistic regression showing no statistically significant difference in December (OR = 1.03; 95 % CI 0.91–1.16) or January (OR = 0.96; 95 % CI 0.85–1.09). A similar (lack of) pattern was observed when “average weekly expenditure on alcohol for own consumption” was analysed as a proxy metric for alcohol consumption. On average, expenditure was 2 % lower in January and comparable to the rest of the year in December, but neither reached statistical significance (P ~ 0.58 and 0.94, respectively); data not shown.

These trends are in agreement with trends in monthly UK alcohol revenue and customs data for that time period [23] and illustrate a stable trend across the year but with a peak in sales during November and December for the festive season (Additional file 1: Figure S2). The following decline in sales in January and February may imply reduced consumption of alcohol which is not observed in our self-reported data, or alternatively may be the result of consumption of alcohol bought for, but left over from the festive period; the latter being in agreement with our results.

## Discussion

These analyses indicate that the frequency of attempts to cut down alcohol consumption in England is significantly higher in January compared to other months of the year. It is likely that this is a reaction to consumption during the December festive period and as a result of New Year’s resolutions.

Our data, however, do not indicate that this increase in attempts to reduce alcohol intake in January was accompanied by a significant change in consumption (at population level); regardless of the metric we use to characterise consumption. The pattern of alcohol consumption is relatively stable across the year, although cumulative consumption is somewhat lower over the summer months.

After appropriate weighting of the samples, the proportion of people who indicated they abstained from alcohol was 30 %, which is about 10 % higher than that proportion in the Health Survey for England (HSE) [24]. One possible explanation is that the ATS asks respondents how often they drink alcohol and classifies people into either ‘never’ or ‘monthly or less’ according to the standard AUDIT questionnaire. Several surveys, including the HSE, ask respondents to clarify whether ‘never’ means ‘never’ or ‘rarely’ and in the case of the HSE respondents answer ‘rarely’ in about a third of cases, which may to some extent explain the observed difference. Additionally, both surveys are based on self-reporting of consumption which may have led to biases in estimated population proportions (because, for example, people with high consumption may under-report or may not participate), and this may have differed between both surveys.

The observed temporal pattern did not correspond to those observed in previous studies undertaken in other countries which reported a peak in consumption in December [4, 6–8, 25]. Our results did mimic, although less obvious in our data, previously reported reduced consumption in the first months of the year [4]. We also observed slightly higher proportion of higher risk drinkers in the spring/early summer, which was previously demonstrated more convincingly in other countries [5–8], and which did not translate into more binge drinking episodes in those months. It is unlikely that these differences could be attributed to issues of self-reporting since although these may affect absolute numbers or proportions, as highlighted above, it is unlikely this would differ substantially from 1 month to the next.

The lack of correlation between the increase in attempts to reduce alcohol consumption in January and measurable change in population alcohol consumption has similarly been observed in primary care patients [12]; although in other studies positive changes in consumption were observed [11].

These analyses indicate that alcohol consumption in England is fairly stable across the year, and that although an increase in attempts to reduce alcohol consumption in January is evident, this is not accompanied by significant change in actual consumption (at least not for long enough to be reported). This has direct implications for initiatives to reduce alcohol consumption in that if these can capitalize on a “natural” surge in motivation and attempts to reduce consumption during the campaign’s running period resulting in sustained motivation, this could result in clear population health benefits.

More specifically given the timing of the effect we observed, these results provide endorsement for Alcohol Concern’s “Dry January” campaign that focusses on continuing support during the whole period aimed at sustained behavioural change. Aside from normal post-festivities attempts, the observed increase in attempts to reduce consumption may to some extent already be related to the “Dry January” campaign [13], but because we have no comparable data from the time period prior to the inception of “Dry January”, we cannot evaluate what the impact of this campaign on top of normal patterns in attempts to reduce consumption could be. Additionally, even if these data were available, “Dry January” has estimated registrations in the order of 50,000 people, which are unlikely to show up in survey data collected at a national level with the sample sizes collected by the ATS. If the uptake of “Dry January” increased substantially, then an evaluation may be possible where lower uptake years were compared to those with higher uptake.

Similarly in primary care, where this does not happen already, these data suggest that January would be a good period to re-iterate the benefits of reducing a patient’s alcohol consumption during general practitioner visits.

In contrast to other studies [3–7, 26], these analyses do not provide much evidence of significant seasonal variation in alcohol consumption patterns, and do not provide additional evidence that this should be taken into account in future studies in this population. However, although this may be correct in this population, alternatively, and in agreement with previous studies, it may also indicate that the AUDIT questions used in ATS are better for detecting harmful levels of drinking [27] in the population than to quantitatively assess temporal patterns of consumption [19, 20].

The strength of the current study is that the ATS methodology is firmly established and based on the longer running Smoking Toolkit Study [15] and that after 23 waves data on over 38,000 respondents, data representative of the general population of England are available. Moreover, the temporal patterns are comparable across the 2 years covered suggesting our inferences are based on stable temporal patterns. Although random location sampling cannot completely exclude the possibility of bias, it is considered superior to simple quota sampling, while additional weighing of the data in the analyses further minimizes residual sampling bias.

Ideally, questions on frequency of drinking events as well as quantity of drinks consumed per event and more irregular episodes of heavy drinking should be included when assessing alcohol consumption [10, 27–29], but in this study we were only able to include two of these (*e.g.* irregular episodes of heavy drinking were not specifically evaluated).

The lack of any significant changes in drinking habits in January, when a significant increase in reported attempts to reduce intake as observed may be correctly inferred, but alternatively the ATS does not include questions specifically referring to current consumption. Instead, they ask about average consumption or ‘typical behaviour during drinking occasions’ and either do not stipulate a specific time frame or refer to the previous 6 months, and may not be sensitive enough to pick up relatively minor changes at population level. The AUDIT tool was developed to screen for hazardous and harmful alcohol consumption and not to accurately reflect population alcohol consumption patterns [20]. Nonetheless, estimates of typical behaviour seem to be, regardless of the actual reference period [8], directly influenced by current or recent behaviour [5, 8]—especially for recent compared to old drinking events [30]. Studies in other populations indicate that the use of AUDIT questions can reflect current drinking habits [31–33] and can be used to detect temporal trends in consumption [34]. Data comparing self-reported consumption for different temporal scales show good correlations [8, 35], and the patterns in our data suggest as much since we observed a reported modest increase in binge drinking events during the festive season followed by a decrease in January and February (as well as during the summer period). These are however, likely biased downwards as a result of the longer reference period [9, 36].

Another possible explanation for the absence of trends in consumption is that participants would have answered the survey at different times during the month, which may have impacted on the ability to detect seasonal changes. Regardless of the specific assessment method used here, problems with the validity of self-reported alcohol consumption are well documented [9, 10, 37, 38]. This is shown by evidence indicating that respondents to alcohol consumption questionnaires refer to the ‘picture of the drinking self’ more than to specific memories of actual drinking events [9]. Because of these uncertainties, an explicit and shorter reference period would have been beneficial [31], although again data suggest that self-reported consumption data with short recall periods are also prone to misclassification [9]. A possible further extension that has been shown to increase the accuracy of reported consumption could be the inclusion of an online component to the questionnaire [39]. Furthermore, population self-reported alcohol consumption is generally under-reported and typically accounts for only 40 to 60 % of total alcohol sales [40]. We nonetheless believe that the metrics used, although likely not sensitive to small changes at population level, would have picked up differences of the order anticipated if attempts to reduce consumption had resulted in a similar change in actual consumption. Future work, ideally not based on self-reported consumption but for example using biomarkers, will be required to investigate the legitimacy of this assumption.

These analyses further focussed on seasonal patterns of alcohol consumption and attempts to reduce consumption at population level only, and potential differences between population subgroups or for specific vulnerable groups in society were not further explored. There is reason to assume that consumption and motivational patterns will differ between subgroups and between regions in the UK (for example: [41, 42]), and future studies can build upon the results of this paper and explore differences between population subgroups.

Finally, in agreement with others [27] we argue that the inclusion of more detailed alcohol questions on drinking patterns and context would be beneficial in assessment of associations between drinking and its consequences.

## Conclusions

This is the first study to explore temporal patterns in alcohol consumption as well as attempts to reduce alcohol intake in England, and indicates that attempts to reduce alcohol consumption are most frequent in January, but that there is no evidence that this is accompanied by significant changes in consumption patterns and quantity of alcohol at population level. These results imply that January would be a good month to initiate population-level interventions, such as the Dry January initiative, provided additional support and follow-up on consumption is included in these programmes, as focussing on motivational change alone is likely not enough.

## Additional file

Additional file 1: Figure S1. ACF and PACF plots for evaluation of residual autocorrelation. **Table S1.** Attempts to reduce alcohol consumption. All months as dummies vs other months. **Figure S2.** UK total alcohol cash receipts per month (HM Revenue and Customs). (PDF 149 kb)
