# Supplementary material for: Temporal patterns of alcohol consumption and attempts to reduce alcohol intake in England
Source: BMC Public Health. 2016 Sep 1;16:917. doi: 10.1186/s12889-016-3542-7 (PMC5009608; doi:10.1186/s12889-016-3542-7)
Supplement: Additional file 1: Figure S1. — ACF and PACF plots for evaluation of residual autocorrelation. Table S1. Attempts to reduce alcohol consumption. All months as dummies vs other months. Figure S2. UK total alcohol cash receipts per month (HM Revenue and Customs). (PDF 149 kb) [file 12889_2016_3542_MOESM1_ESM.pdf]

Temporal patterns of alcohol consumption and attempts to reduce alcohol intake in England

Frank de Vocht[1,2,*], Jamie Brown[1,3], Emma Beard[1,3], Colin Angus[1,4], Alan Brennan[1,4], Susan Michie[1,3], Rona Campbell[1,2], Matthew Hickman[1,2]

[1]: NIHR School for Public Health Research (SPHR).

[2]: School of Social and Community Medicine, University of Bristol, Bristol, UK

[3]: Department of Clinical, Educational and Health Psychology, University College London, London, UK.

[4]: ScHARR, School of Health and Related Research, University of Sheffield, Sheffield, UK

Online Supplementary Material

This work was funded by the National Institute for Health Research School for Public Health Research (NIHR SPHR). The views expressed are those of the author(s) and not necessarily those of the NHS, the NIHR or the Department of Health. NIHR SPHR is a partnership between the Universities of Sheffield, Bristol, Cambridge, Exeter, UCL; The London School for Hygiene and Tropical Medicine; the LiLaC collaboration between the Universities of Liverpool and Lancaster and Fuse; The Centre for Translational Research in Public Health, a collaboration between Newcastle, Durham, Northumbria, Sunderland and Teesside Universities. The views expressed are those of the author(s) and not necessarily those of the NHS, the NIHR or the Department of Health.

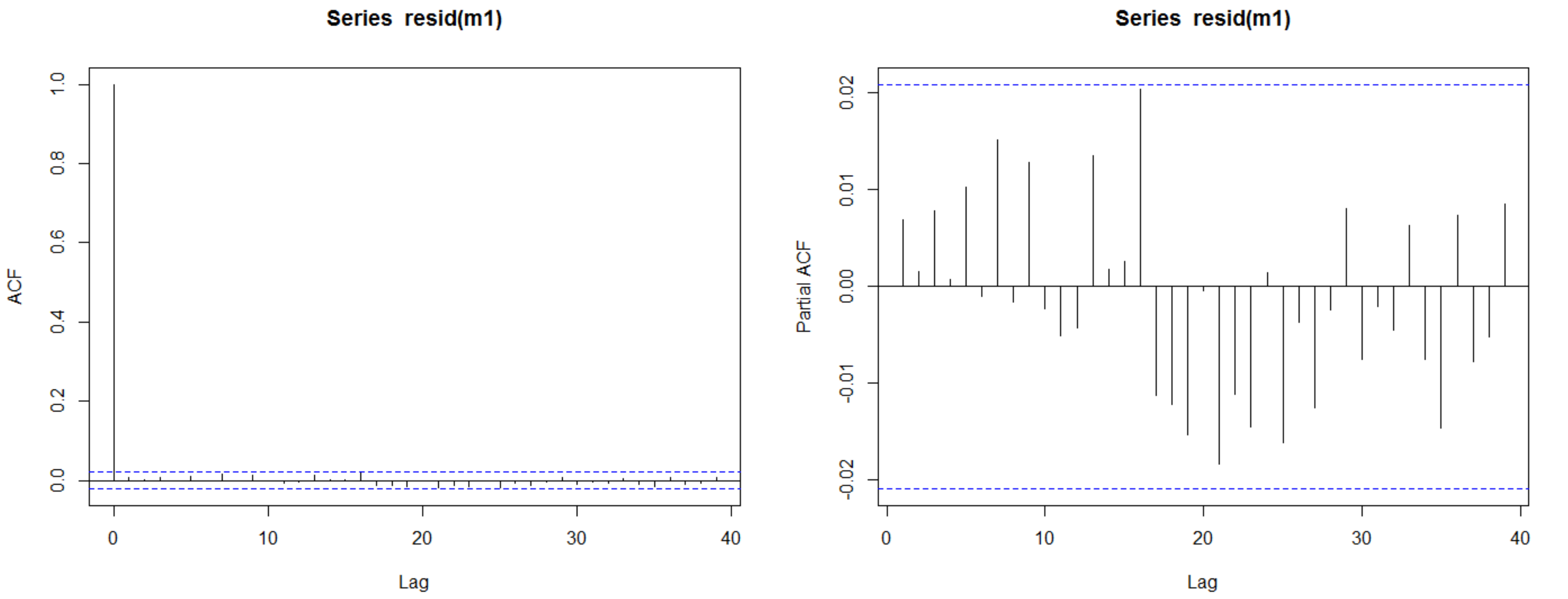

Figure Online Supplementary Material S1. ACF and PACF plots for evaluation of residual autocorrelation.

Table Online Supplementary Material S1. Attempts to reduce alcohol consumption. All months as dummies vs other months.

	Odds Ratio* (95% CI)	P value
Reference (all other months)	1	
January	1.41 (1.16-1.73)	0.001
Feb	1.08 (0.81-1.42)	0.606
March	0.91 (0.74-1.12)	0.377
April	0.96 (0.80-1.17)	0.704
May	1.04 (0.85-1.28)	0.672
Jun	0.88 (0.71-1.10)	0.270
July	1.10 (0.90-1.34)	0.355
Aug	0.79 (0.64-0.96)	0.017
Sep	1.07 (0.87-1.30)	0.526
Oct	1.02 (0.84-1.24)	0.841
Nov	1.07 (0.87-1.31)	0.536
December	0.80 (0.65-1.00)	0.050
January 2015	1.41 (1.07-1.85)	0.014
January 2016	1.38 (1.04-1.83)	0.024

*: adjusted for time, age, sex, social class, education and occupation

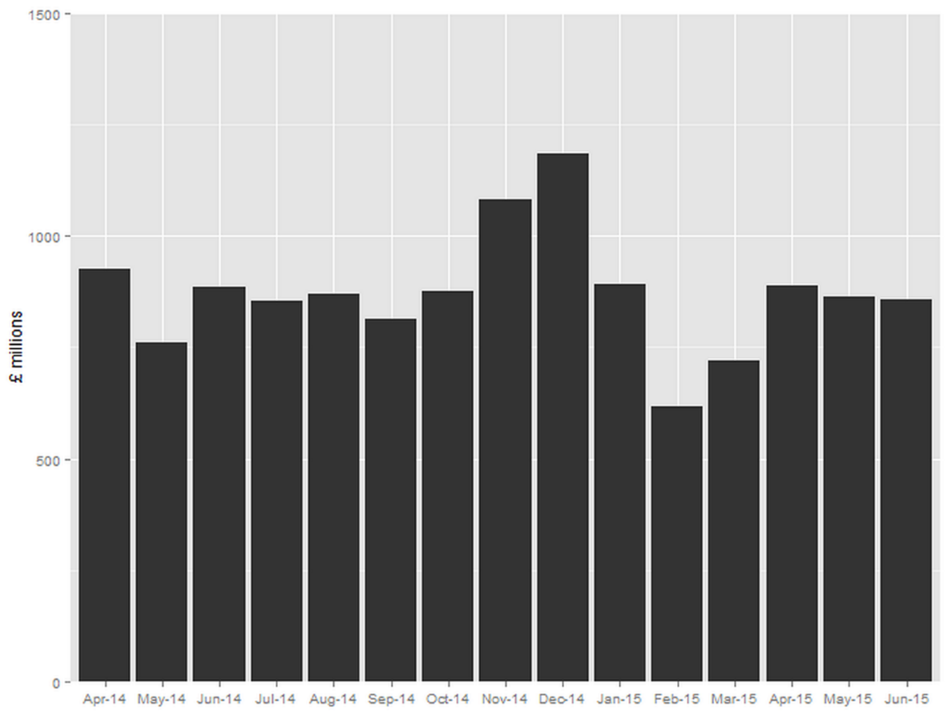

Figure Online Supplementary Material Figure S2. UK total alcohol cash receipts per month (HM Revenue and Customs)
